# 1,2,3,4,6-Penta-*O*-Galloyl-Beta-D-Glucopyranoside Inhibits Proliferation of Multiple Myeloma Cells Accompanied with Suppression of MYC Expression

**DOI:** 10.3389/fphar.2018.00065

**Published:** 2018-02-02

**Authors:** Duurenjargal Tseeleesuren, Rajni Kant, Chia-Hung Yen, Hui-Hua Hsiao, Yi-Ming A. Chen

**Affiliations:** ^1^Graduate Institute of Medicine, College of Medicine, Kaohsiung Medical University, Kaohsiung, Taiwan; ^2^Center for Infectious Disease and Cancer Research, Kaohsiung Medical University, Kaohsiung, Taiwan; ^3^Graduate Institute of Natural Products, College of Pharmacy, Kaohsiung Medical University, Kaohsiung, Taiwan; ^4^Department of Medical Research, Kaohsiung Medical University Hospital, Kaohsiung, Taiwan; ^5^Research Center for Natural Products and Drug Development, Kaohsiung Medical University, Kaohsiung, Taiwan; ^6^Division of Hematology-Oncology, Department of Internal Medicine, Kaohsiung Medical University Hospital, Kaohsiung, Taiwan; ^7^Faculty of Medicine, Kaohsiung Medical University, Kaohsiung, Taiwan; ^8^Institute of Biomedical Sciences, National Sun Yat-sen University, Kaohsiung, Taiwan

**Keywords:** multiple myeloma, MYC, PGG, DEPTOR, G1 arrest, apoptosis, proteasome-inhibitors, JQ1

## Abstract

Multiple myeloma (MM) still remains an incurable disease, therefore discovery of novel drugs boosts the therapeutics for MM. The natural compound 1,2,3,4,6-Penta-*O*-galloyl-beta-D-glucopyranoside (PGG) has been shown to exhibit antitumor activities against various cancer cells. Here, we aim to evaluate antitumor effects of PGG on MM cell lines. PGG inhibited the growth of three different MM cell lines in a dose- and time-dependent manner. Cell cycle analysis revealed that PGG treatment caused cell cycle arrest in G1 phase. It also induced apoptosis which was indicated by significant increases of Annexin V positive cells, caspase 3/7 activity, and cleaved caspase 3 expression in PGG treated MM cell. Since MYC is frequently hyperactivated in MM and inhibition of MYC leads to MM cell death. We further demonstrated that PGG decreased MYC expression in protein and mRNA levels and reversed the mRNA expression of MYC target genes such as p21, p27, and cyclin D2. In addition, PGG also reduced protein expression of DEPTOR which is commonly overexpressed in MM. Unexpectedly, PGG antagonized the cytotoxic effect of bortezomib in the combination treatment. However, PGG treatment sensitized MM cells to another proteasome inhibitor MG132 induced cytotoxicity. Moreover, MYC inhibitor JQ1 enhanced the cytotoxic effect of bortezomib on MM cells. Our findings raised concerns about the combinatory use of bortezomib with particular types of chemicals. The evidence also provide useful insights into the combination of MYC and proteasome-inhibitors for MM therapy. Finally, PGG has a therapeutic potential for treatment of MM and further development is mandatory.

## Introduction

Multiple myeloma (MM) is a malignant B cell disorder arising from plasma cells and accumulating in the bone marrow leading to osteolytic bone lesions and impaired hematopoiesis (Tosi, 2013; Follin-Arbelet et al., 2015). During the past few decades, there have been major advances in the treatment of MM which have improved the overall survival of the patients. The common treatment for MM is chemotherapy with or without autologous stem cell transplantation. The induction therapy using lenalidomide, thalidomide, or bortezomib plus autologous stem cell transplantation is the standard treatment for patients below 65 years of age (Moreau et al., 2015). Transplantation is unsuitable for most of the patients older than 65. Instead, chemotherapy, a combination of melphalan and prednisone with thalidomide, lenalidomide, or bortezomib is a standard treatment for the patients aged over 65 of age (Facon et al., 2007; Palumbo et al., 2008; San Miguel et al., 2008). The majority of MM patients are relapsed after the application of currently available therapeutic options, and then all of them will eventually develop resistant or refractory disease (Orlowski, 2013). Moreover, drugs used for MM treatment have serious side effects and cost of these drugs is very high (Gay and Palumbo, 2010). Thus, in view of treatment limitations, there is great urgency to find additional or substitute drugs (aiming to reduce side effects, drug resistance, expenses, and inefficiency of existing MM therapies) for MM treatment.

DEPTOR (DEP domain-containing mTOR-interacting protein), an mTOR interacting protein inhibits mTOR activity and its expression is usually down-regulated in most tumors. Conversely, DEPTOR is immensely up-regulated in a subset of MM cells containing c-MAF/MAFB or cyclin D1/D3 translocations. Higher DEPTOR expression in these MM cells are required to sustain PI3K/mTORC2/Akt activation and survival. Furthermore, reduction of DEPTOR levels leads to apoptosis in MM cells (Peterson et al., 2009). In addition, DEPTOR silencing increases chemo sensitivity to doxorubicin and melphalan in RPMI 8226 MM cells (Zhang H. et al., 2013; Zhang H.R. et al., 2013). Therefore, DEPTOR is an attractive therapeutic target for MM.

In addition to DEPTOR, the proto-oncogene MYC is highly dysregulated in MM and is a key contributor in MM development. The MYC rearrangement was found in nearly half of MM patients and became one of the most common mutated gene in MM (Affer et al., 2014; Holien et al., 2015). Various reports showed that the activity of MYC increases with disease stages and MYC overexpression was related to poor prognosis in MM patients (Chng et al., 2011). Increasing studies confirming the importance of MYC overexpression in MM, which makes MYC an attractive target for developing novel MM therapeutic modalities.

**FIGURE 1:**
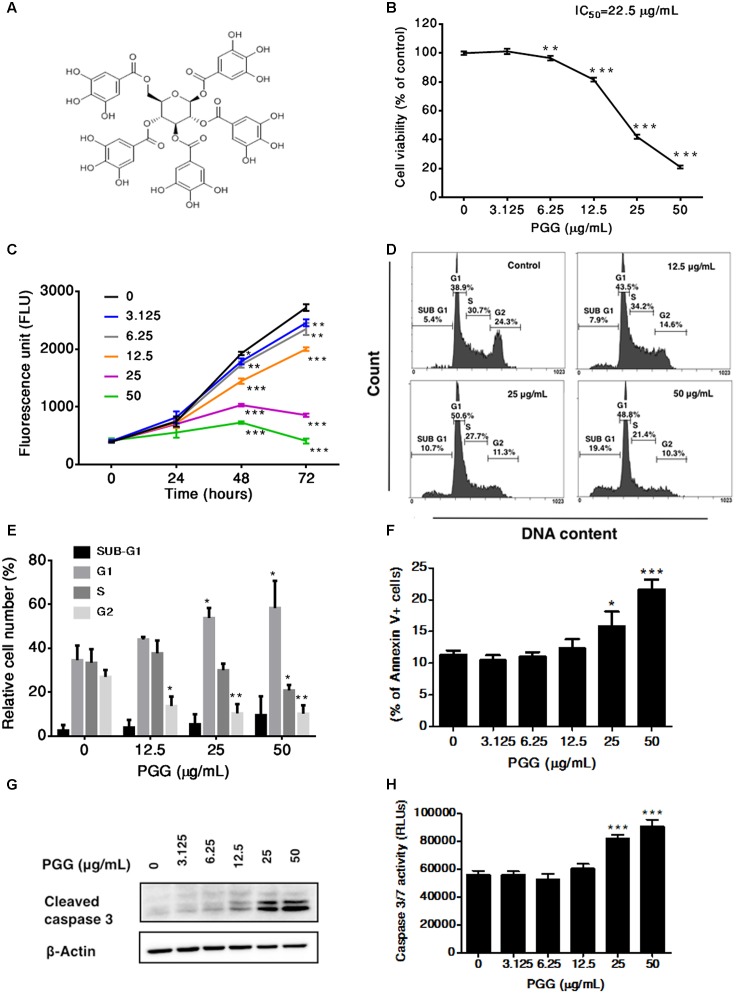
Effect of PGG treatment on growth, cell cycle progression and induction of apoptosis in RPMI 8226 cells. **(A)** Chemical structure of 1,2,3,4,6-penta-*O*-galloyl-beta-D-glucopyranoside. **(B)** The viability of RPMI 8226 cells after 72 h of PGG treatment determined by the alamarBlue^®^ assay as described in Section “Materials and Methods.” The percentage of viable cells normalized to solvent treated control were plotted. IC_50_ value of PGG is shown in the figure. Results are as means ± SD (*n* = 3). **(C)** RPMI 8226 cells were treated with indicated doses of PGG. Cell proliferation was evaluated at 0, 24, 48, and 72 h using alamarBlue^®^ assay. Results are as means ± SD (*n* = 3). **(D)** Representative images and **(E)** quantitative data indicates percentage of the cells at different phases of cell cycle in RPMI 8226 cells after 24 h of PGG treatment as assessed by DNA flow cytometric analysis. Data presented as means ± SD (*n* = 3). **(F)** RPMI 8226 cells were cultured with indicated doses of PGG for 24 h, stained with Annexin V-FITC and analyzed by flow cytometry. The graph represents the percentages of annexin V+ apoptotic cells. Data presented as means ± SD (*n* = 3). **(G)** RPMI 8226 cells were treated with indicated doses of PGG for 24 h and appearance of cleaved form of caspase-3 was determined by immunoblot analysis. Expression of β-actin was used as a loading control. **(H)** The activation of caspase 3/7 was observed in RPMI 8226 cells after 24 h of PGG treatment. Data presented as means ± SD (*n* = 3). ^∗∗∗^*P* < 0.001, ^∗∗^*P* < 0.01, ^∗^*P* < 0.05 (Student’s *t*-test).

Historically, natural products or their derivatives have contributed to the majority of novel drugs or drug leads (Lahlou, 2013). 1,2,3,4,6-penta-*O*-galloyl-beta-D-glucopyranoside (PGG) is a naturally occurring polyphenolic (**Figure 1A**) found in a number of medicinal herbs and plants such as *Rhus chinensis Mill*, *Paeonia lactiflora*, *Schinus terebinthifolius*, etc. (Cavalher-Machado et al., 2008; Yu et al., 2011; Kant et al., 2016). It has been demonstrated that PGG exhibits an anticancer effect in various cancers, such as lung cancer (Huh et al., 2005), prostate cancer (Hu et al., 2008), breast cancer (Chen et al., 2003), liver cancer (Oh et al., 2001), and sarcoma (Miyamoto et al., 1987). Mechanistic studies in the different cancer cell models reported that PGG modulates various cellular processes including apoptosis, angiogenesis, metastasis, and signaling pathways (Zhang et al., 2009). A few reports also showed that reactive oxygen species and autophagy-dependent senescence were induced by PGG in various cancer cell lines (Dong et al., 2014). Moreover, in hematological malignancies, PGG has shown antitumor effects on different types of leukemia. Pan et al. (1999) previously showed that PGG could effectively inhibit human promyelocytic leukemia HL-60 cells growth and induce apoptosis. In addition, *in vivo* studies have shown that PGG enhances the antitumor activity of imatinib in chronic myelogenous leukemia K562 cells in mice (Kwon et al., 2012). Nevertheless, the antitumor effects of PGG against multiple myeloma have not been reported. Most importantly, recent studies from our lab identified PGG as a potent MYC inhibitor. PGG inhibits MYC transcription and promotes MYC degradation through proteasome independent pathway (Kant, 2017, unpublished data). Therefore, this study aimed to evaluate the therapeutic potential of PGG in MM and the effects of PGG alone and/or in combination with clinically used MM therapeutic agent bortezomib in myeloma cell lines. We also studied the effect of PGG on DEPTOR expression in MM cell lines.

## Materials and Methods

### Cell Culture and Reagents

Human MM cell lines RPMI 8226 and U266B1 were cultured in RPMI 1640 media supplemented with 10% fetal bovine serum (FBS) (HyClone, Logan, UT, United States), L-glutamine (2 mM), streptomycin (100 μg/mL), penicillin (100 U/mL), and non-essential amino acids (0.1 mM). Besides, NCI-H929 was cultured in RPMI 1640 media supplemented with 10% FBS, streptomycin (100 μg/mL), penicillin (100 U/mL), HEPES (10 mM), sodium pyruvate (1 mM), glucose (4.5 g/L), L-glutamine (2 mM), and 2-mercaptoethanol (0.05 mM). All cell lines were maintained at 37°C in a humidified CO2 incubator and passaged every 4–5 days. The bortezomib, JQ1 and MG132 were obtained from Sigma-Aldrich (St. Louis, MO, United States) and PGG was prepared as described previously (Kant et al., 2016).

### Cell Viability and Cell Proliferation Assay

AlamarBlue^®^ assay (AbD Serotec, Oxford, United Kingdom) was used to assess cell viability and cell proliferation according to the manufacturer’s instructions. In brief, RPMI 8226, U266B1, and NCI-H929 cells were seeded in 96 well plates (1 × 10^4^ cells/well) and exposed to various concentrations of PGG (3.125 to 50 μg/mL), bortezomib (3.125 to 50 nM) and combinations. MG132 was tested at concentrations from 0.0022 to 0.625 μM alone and in combinations with PGG (3.125 to 50 μg/mL), whereas JQ1 was tested at concentrations from 62.5 to 1000 nM alone and in combinations with bortezomib (3.125 to 50 nM). After 72 h of incubation at 37°C in a 5% CO_2_ atmosphere, alamarBlue^®^ was added and further incubated for 4 h to assess the cytotoxic effect of the tested drugs. Blank wells (no cells) containing the same amount of test substance were used for background subtraction and the relative number of viable cells were expressed as the percentage of the mean of the relative solvent controls. For proliferation assay, RPMI 8226 cells were seeded and treated with PGG as mentioned above. At 0, 24, 48, and 72 h’ time points, alamarBlue^®^ was added and further incubated for 4 h at 37°C. Fluorescence of the reduced alamarBlue^®^ was measured using a Synergy HT Multi-Mode Microplate Reader (Biotek Instruments, Winooski, VT, United States) in both assays.

### Cell Cycle Analysis

For cell cycle analysis RPMI 8226 cells were seeded and treated with PGG (3.125 to 50 μg/mL) for 24 h. After 24 h of treatment, cells were collected, washed and fixed by adding 70% ethanol at 4°C overnight. Ethanol-suspended cells were centrifuged, washed to remove residual ethanol, resuspended in 100 μg/mL RNaseA for 1.5 h at 37°C and incubated with 1 mg/mL propidium iodide for 15 min at room temperature. The cells cycle distribution was analyzed by flow cytometer (Accuri C6, BD Biosciences).

### Annexin V Assay

Annexin V-FITC Apoptosis Detection Kit (eBioscience, San Diego, CA, United States) was used to stain and evaluate annexin V positive cells in control-treated and PGG-treated RPMI 8226 cells according to the manufacturer’s protocol. In brief, following PGG treatment, 1 × 10^6^ cells were pelleted, washed and resuspended in binding buffer. Subsequently, Annexin V-FITC was added to the cells, incubated in the dark for 20 min at room temperature and analyzed by Flow cytometer (Accuri C6, BD Biosciences). Data analysis was also conducted using BD Accuri C6 Software.

### Caspase 3/7 Fluorescence Assay

RPMI 8226 cells were treated with solvent or PGG for 24 h. Caspase 3/7 activity was measured by Apo-ONE^®^ Homogeneous Caspase-3/7 assay kit (Promega) according to the manufacturer’s protocol.

### Immunoblotting

Cells were harvested for lysate preparation by using RIPA buffer containing protease inhibitor cocktail (Roche Molecular Biochemicals) and phosphatase-inhibitors (10 mM Na_3_VO_4_, 1 mM NaF, 5 mM NaPPi). Protein concentrations were quantified by Bio-Rad Protein Assay (Bio-Rad) and immunoblotting was carried out as described previously (Yen et al., 2012). The antibodies: anti-MYC (D84C12, Cell Signaling Technology), anti-caspase-3 (9H19L2, Thermo scientific), anti-DEPTOR (D9F5, Cell Signaling Technology), anti-GAPDH (Sigma-Aldrich) and anti-β-actin (AC-15, Sigma-Aldrich) were used according to the description in manufacturer’s instructions.

### Quantitative Real-Time PCR (qRT-PCR)

The RNA was isolated by using Trizol reagent (Life Technologies) according to the manufacturer’s protocol. Cellular RNA was reverse transcribed into complementary DNA using a SuperScript II RNase H-Reverse Transcriptase Kit (Life Technologies). PCR reactions were carried out using a Super Script II Reverse Transcriptase Kit (Invitrogen, Inc., Carlsbad, CA, United States) on an Applied Biosystems Step-One-plus real-time PCR system (Applied Biosystems, Foster City, CA, United States). The mRNA level of TATA-box binding protein (TBP) was used as an internal control. The primer pairs used for qRT-PCR were: TBP forward, 5′-TGCACAGGAGCCAAGAGTGAA-3′ and reverse, 5′-CACATCACAGCTCCCCACCA-3′; MYC forward: 5′-TTCGGTTGTTGCTGATCTGTCT-3′ and reverse: 5′-CCCTCCACTCGGAAGGACTAT-3′; DEPTOR forward: 5′-CAGGAATGAAGGTCTGTCAGTTTG-3′ and reverse: 5′-TGCTCACGGTCCGGTAGTCTA-3′; p21 forward: 5′-TGAGCCGCGACTGTGATG-3′ and reverse: 5′-GTCTCGGTGACAAAGTCGAAGTT-3′; p27 forward: 5′-TGCAACCGACGATTCTTCTACTCAA-3′ and reverse: 5′-CAAGCAGTGATGTATCTGATAAACAAGGA-3′; cyclin D2 forward: 5′-CTGTGTGCCACCGACTTTAAGTT-3′ and reverse: 5′-GATGGCTGCTCCCACACTTC-3′.

### Statistical Analysis

GraphPad Prism 5.0 software (La Jolla, CA, United States) and MS excel were used for statistical analyses of all the data. To compare groups, *P*-values were determined by student’s two- sided *t*-tests and considered to be statistically significant at *P* < 0.05.

## Results

### PGG Induces G1 Arrest and Apoptosis in RPMI 8226 Cells

To determine the therapeutic potential of PGG against MM, we first evaluated the inhibitory effect of PGG on the growth of RPMI 8226 human myeloma cells. PGG suppressed the proliferation of RPMI 8226 cells in a dose-dependent manner with IC_50_ 22.5 μg/mL after 72 h of treatment (**Figure 1B**). Subsequently, 24, 48, and 72 h of exposure to PGG treatment significantly inhibited the proliferation of RPMI 8226 cells in a time-dependent manner (**Figure 1C**). Next, to investigate the growth inhibitory mechanism of PGG on MM cells, we evaluated the effect of PGG treatment on cell cycle progression. Enhanced accumulation of RPMI 8226 cells in the G1 phase of cell cycle was observed after treatment with PGG in a dose-dependent manner with a concomitant decrease in S-phase cells. Moreover, an increase of Sub-G1 population indicated induction of apoptosis, after PGG treatment (**Figures 1D,E**). To confirm whether PGG treatment induces apoptosis in RPMI 8226 cells, we next observed apoptosis induction by Annexin V assay. Consistent with the anti-proliferative effect of PGG, notable increases of Annexin V positive cells were observed in a dose-dependent manner (**Figure 1F**). Furthermore, increased levels of cleaved caspase-3 were detected after the treatment with indicated doses of PGG for 24 h (**Figure 1G** and Supplementary Figure S3). In addition, dose-dependent increase in caspase 3/7 activity was observed in these cells after PGG treatment (**Figure 1H**). Taken together PGG caused apoptosis in MM cells.

### PGG Treatment Inhibits MYC and DEPTOR Expression in RPMI 8226 Cells

**FIGURE 2:**
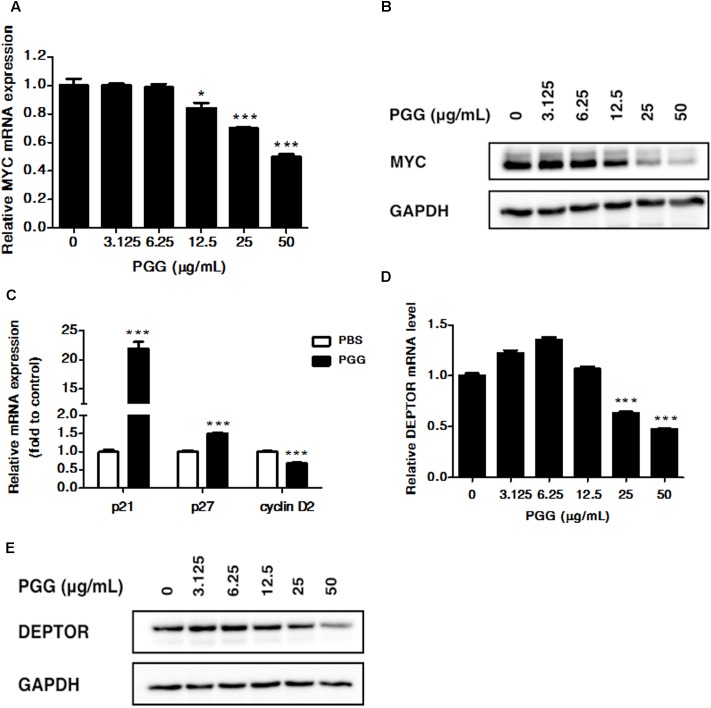
PGG inhibits MYC and DEPTOR expression. **(A,B)** RPMI 8226 cells were treated with indicated doses of PGG for 24 h. mRNA **(A)** and protein **(B)** expression of MYC was detected by quantitative real-time PCR (qRT-PCR) and immunoblotting assay. Results are means ± SD (*n* = 3). GAPDH expression was used as loading control. **(C)** The mRNA levels of MYC target genes (p21, p27, and cyclin D2) after 24 h of PGG treatment in RPMI 8226 cells were detected by qRT-PCR. Data presented as fold to solvent control. The graph shows the means ± SD (*n* = 3). Effect of PGG on DEPTOR mRNA **(D)** and protein **(E)** expression in RPMI 8226 cells after 24 h of treatment. GAPDH expression was used as a loading control. Results are the means ± SD (*n* = 3). ^∗∗∗^*P* < 0.001, ^∗^*P* < 0.05 (Student’s *t*-test).

Recently, our group showed that PGG inhibits MYC expression in hepatocellular carcinoma (Kant, 2017, unpublished data). Moreover, the progression of MM is related to the increased MYC. Therefore, in order to determine whether PGG exerts its effect through MYC inhibition in MM, we examined the effect of PGG treatment on mRNA and protein expression of MYC in RPMI 8226 cells. As shown in **Figures 2A,B** and Supplementary Figure S3, PGG treatment inhibits MYC mRNA and protein expression in a dose-dependent manner. Moreover, we evaluated the effect of PGG on MYC function by analyzing expression of MYC target genes after PGG treatment. Consistent with MYC inhibition PGG treatment increases mRNA expression of p21, p27 and decreases expression of cyclin D2 in RPMI 8226 cells (**Figure 2C**). In addition to MYC, DEPTOR is also overexpressed in MM and its inhibition has been reported to have therapeutic potential in MM. Therefore, we also tested whether PGG treatment could inhibit the expression of DEPTOR in RPMI 8226 cells. Results of qPCR and western blot revealed that PGG reduced DEPTOR mRNA and protein expression in a dose-dependent manner in RPMI 8226 cells (**Figures 2D,E** and Supplementary Figure S3).

### Effect of PGG Treatment on NCI-H929 and U266B1 Cells

**FIGURE 3:**
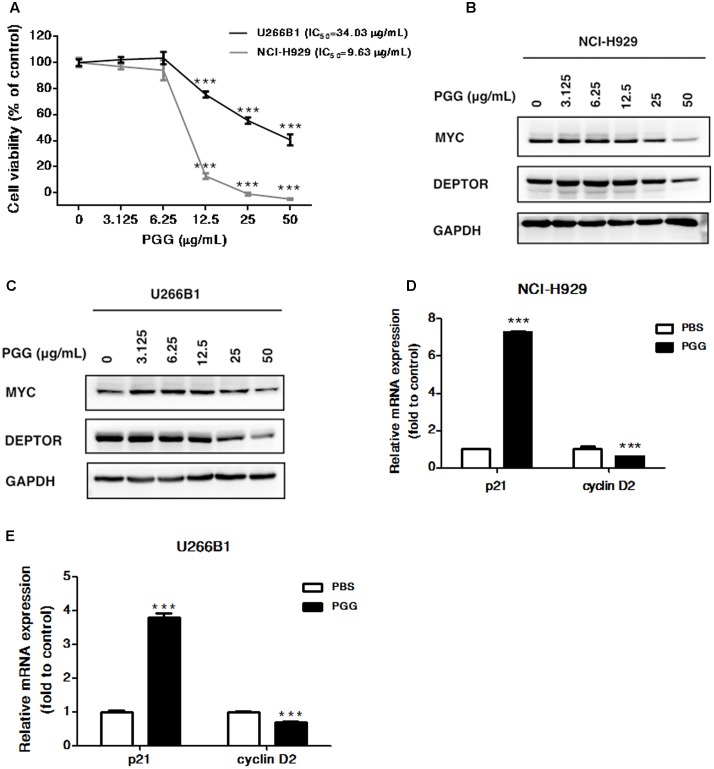
Effect of PGG treatment on NCI-H929 and U266B1 cells **(A)** The viability of NCI-H929 and U266B1 cells were analyzed after PGG treatment for 72 h and determined by alamarBlue^®^ assay as described in Section “Materials and Methods.” IC_50_ value of PGG is shown in the figure. Results are means ± SD (*n* = 3). MYC and DEPTOR protein expression in NCI-H929 **(B)** and U266B1 **(C)** cells treated with indicated concentrations of PGG for 24 h determined by immunoblot assay. GAPDH expression was used as a loading control. The upregulation of p21 and inhibition of cyclin D2 mRNA expression in NCI-H929 **(D)** and U266B1 **(E)** cells treated with PGG for 24 h determined by qRT-PCR. ^∗∗∗^*P* < 0.001 (Student’s *t*-test).

Because, PGG inhibited proliferation of RPMI 8226 cells, therefore, next we sought to further validate the anti-myeloma potential of PGG by using NCI-H929 and U266B1 myeloma cells. As shown in **Figure 3A**, 72 h of PGG treatment dose-dependently inhibited the growth of both cell lines with IC_50_ of 9.63 μg/mL for NCI-H929 and 34.03 μg/mL for U266B1. Subsequently, we examined the effect of PGG treatment on MYC and DEPTOR. As expected, PGG treatment decreased the MYC and DEPTOR protein levels in both MM cell lines in a dose-dependent manner (**Figures 3B,C** and Supplementary Figure S3). Moreover, we also examined the effect of PGG treatment on MYC target genes and found that PGG increases the mRNA expression of p21 and decreases the mRNA expression of cyclin D2 in both cell lines (**Figures 3D,E**). PGG reduced MYC and DEPTOR expression in all tested MM cell lines. Collectively, PGG mediated inhibition of both proteins plays a major role in anti-myeloma activity of PGG.

### PGG Neutralizes the Effect of Bortezomib and Sensitizes the Effect of MG132 to Inhibit MM Cells Growth

**FIGURE 4:**
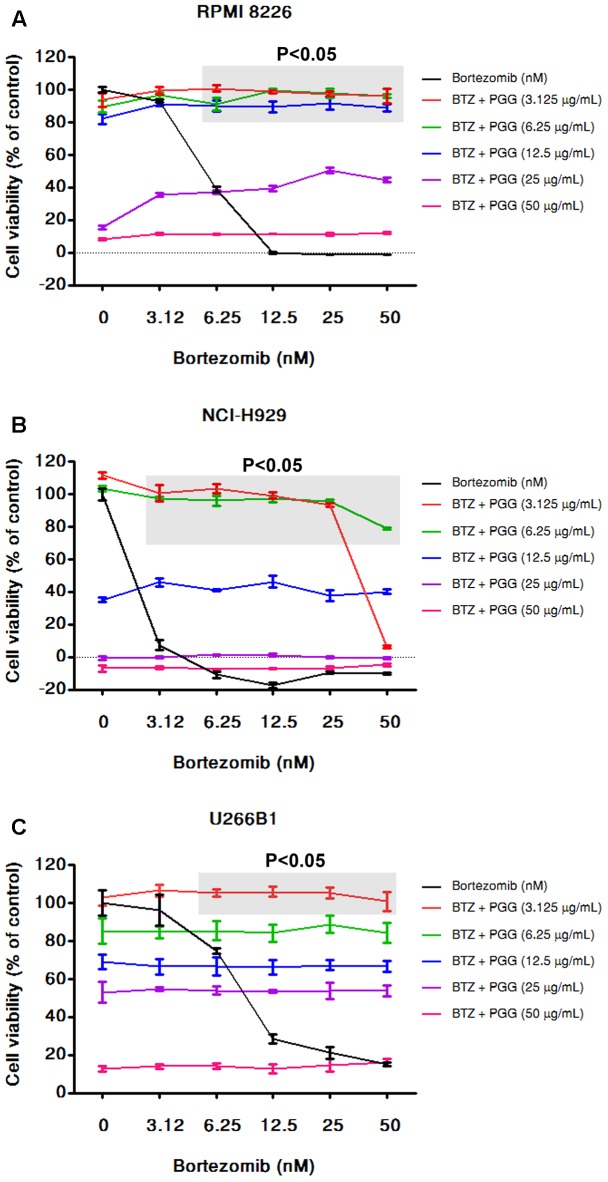
PGG neutralizes the effect of bortezomib in MM cells. RPMI 8226 **(A)**, NCI-H929 **(B)**, and U266B1 **(C)** MM cells were treated with various dosages of PGG (3.125–50 μg/mL) and bortezomib (3.125–50 nM) both alone and in combinations. After 72 h cell viability was assessed by alamarBlue^®^ assay as mentioned in Section “Materials and Methods.” The percentage of viable cells normalized to solvent treated control cells were plotted after background subtraction. The light gray boxes with (*P* < 0.05) represents that the low doses of PGG significantly antagonizing the effect of bortezomib on cell viability. Results are means ± SD (*n* = 3).

**FIGURE 5:**
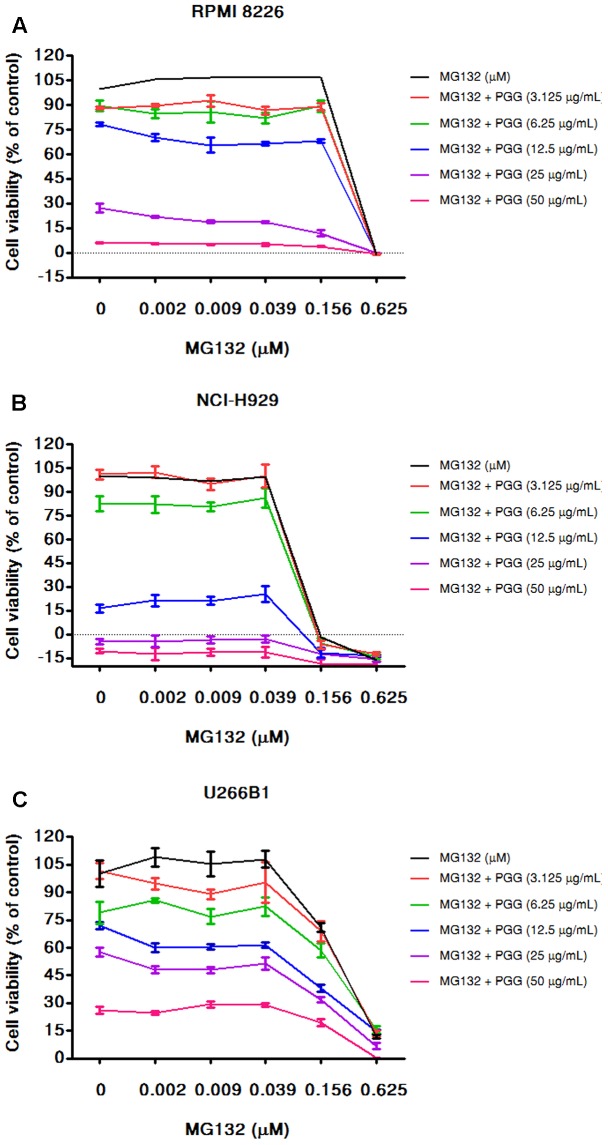
PGG sensitizes the effect of MG132 to inhibit MM cells growth. RPMI 8226 **(A)**, NCI-H929 **(B)**, and U266B1 **(C)** cells were cultured with the indicated concentrations of PGG (3.125–50 μg/mL) and MG132 (0.0022–0.625 μM) both alone and in combinations for 72 h. Cell viability was assessed by alamarBlue^®^ assay and presented as described in Section “Materials and Methods.” The percentage of viable cells normalized to solvent treated control were plotted after background subtraction. Results are means ± SD (*n* = 3).

Bortezomib is a first-class FDA-approved proteasome inhibitor for the treatment of relapsed and refractory MM (Chen et al., 2011). However, adverse effects and resistance to bortezomib treatment is observed in MM patients (Abdi et al., 2013). The combination of bortezomib with different agents has shown beneficial effects (Kapoor et al., 2012; Moreau et al., 2012). Furthermore, the activation of MYC and DEPTOR is well-known to promote growth and survival of MM cells. Thus, it is worthy to evaluate whether PGG (due to its inhibitory effect on MYC and DEPTOR) enhance the efficacy of bortezomib for MM treatment. Conversely, results showed that bortezomib had no combinatorial effect with PGG in all MM cell lines. Surprisingly, low doses of PGG (3.125, 6.25, and 12.5 μg/mL) almost completely neutralizes the bortezomib-induced growth inhibition. Still, we can observe a dose-dependent anti-myeloma effect of PGG in the presence of bortezomib (**Figures 4A–C** and Supplementary Tables S1–S3). Next, to assess whether blocking of anti-myeloma activity of bortezomib by PGG is extended to other proteasome inhibitors or specific to bortezomib only, we evaluated the effect of PGG on MG132 mediated cytotoxicity to MM cells. Interestingly, we observed that PGG treatment did not neutralize MG132 mediated anti-myeloma cytotoxicity. Moreover, the anti-myeloma cytotoxic effect of the combined treatment was greater than that in single treatment at most concentrations tested in RPMI 8226 and U266B1 cells. A few combined concentrations showed the effect of PGG or MG132 only (**Figures 5A–C** and Supplementary Tables S4–S6)

### BET Inhibitor JQ1 Enhances Cytotoxicity Induced by Bortezomib in MM Cells

**FIGURE 6:**
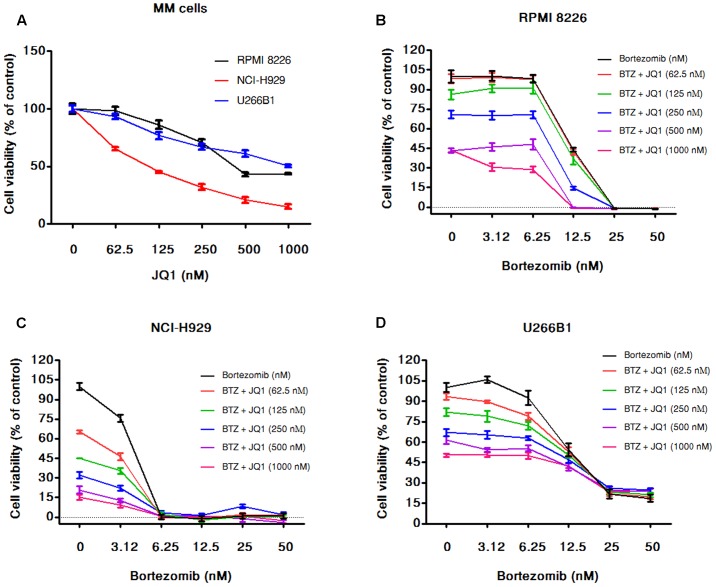
JQ1 enhances cytotoxicity induced by bortezomib in MM cells. **(A)** The viability of RPMI 8226, NCI-H929, and U266B1 cells after 72 h of JQ1 treatment determined by the alamarBlue^®^ assay as described in Section “Materials and Methods.” The percentage of viable cells normalized to solvent treated control were plotted after background subtraction. Results are as means ± SD (*n* = 3). RPMI 8226 **(B)**, NCI-H929 **(C)**, and U266B1 **(D)** cells were incubated with various concentrations of bortezomib (3.125–50 nM) alone and in combination with various concentration of JQ1 (0–1000 nM). After 72 h of the treatments cell viability was assessed by alamarBlue^®^ assay as mentioned above. The percentage of viable cells normalized to solvent treated control cells were plotted after background subtraction. Results are means ± SD (*n* = 3).

In the combination experiments of bortezomib with PGG, we found that PGG neutralized the anti-myeloma efficacy of bortezomib. As a small-molecule compound JQ1 demonstrated repression of MYC expression in MM (Delmore et al., 2011), therefore, next we used JQ1 to assess combination potential of MYC inhibition with bortezomib against MM cell growth. As shown in **Figure 6A**, JQ1 treatment resulted in dose-dependent inhibition of all the MM cell lines tested. As expected, the combination of JQ1 and bortezomib showed increased inhibition of MM cells growth at most of the concentrations tested and few combined concentrations showed the effect of single agent only. In NCI-H929 all cells died at higher concentration of bortezomib therefore, we can see the combination effect at the lowest concentration of bortezomib tested (**Figures 6B–D** and Supplementary Tables S7–S9). Overall this data supports the usefulness of MYC inhibitory compounds with bortezomib for enhancing anti-myeloma efficacy.

## Discussion

For Regardless of the significant advances in treatment of multiple myeloma and patient survival during the past years, the majority of patients still relapse and become resistant to therapy (Mohty et al., 2012). In addition, chemotherapeutic agents used for multiple myeloma therapy have adverse side effects (Gay and Palumbo, 2010). Hence, novel and less toxic agents for multiple myeloma therapy are urgently desired.

PGG, a prototypical gallotannin isolated from plants is well-known for its potent antitumor activities. PGG has shown to alter cell cycle related proteins and induced potent growth inhibition of leukemic cancer cells *in vitro*. Moreover, PGG has a promising safety profile in animal systems (Zhang et al., 2009). In addition, PGG is not mutagenic in the Ames test and possess potent anti-mutagenic activity (Abdelwahed et al., 2007; Kant et al., 2016). Therefore, in the present study, we tested the therapeutic potential of PGG in multiple myeloma. Here, we show that PGG exhibits the potent anti-proliferative effect on three different MM cell lines (RPMI 8226, NCI-H929, and U266B1) in a dose-dependent manner. The IC_50_ for NCI-H929 was 9.63 μg/mL, which is most sensitive to PGG treatment as compared with 22.5 μg/mL for RPMI 8226 and 34.03 μg/mL for U266B1. Furthermore, we observed that PGG treatment resulted in the G1 – phase arrest, along with an apoptosis induction in RPMI 8226 cells. These results are consistent with previous studies on the effect of PGG in different cancer cell lines. Chen and Lin (2004) reported that PGG induces G1 arrest and apoptosis in human Jurkat T (acute T cell leukemia) cells through inhibiting proteasome activity and elevating p27 (Kip), p21 (Cip), and Bax proteins. Pan et al. (1999) also showed similar results that PGG induced apoptosis in human promyelocytic leukemia HL-60 cells. Our results further confirmed these findings in MM cells. The three myeloma cell lines used in this study can be distinguished on the basis of their diverse characteristics (Supplementary Table S10) and might be reflective of the range of sensitivity to PGG. The expression of CD38 and M-CSF were negatively correlated with IC_50_ of PGG while, the expression of TNF-α and PCA-1 were positively associated with IC_50_ of PGG. Importantly, CD38 is highly overexpressed on malignant cells in MM and has been proven to be an attractive therapeutic target for the treatment of MM (Lin et al., 2004; Stevenson, 2006; de Weers et al., 2011). Therefore, we further examined the effect of PGG on CD38 expression and found that PGG treatment inhibited CD38 mRNA expression dose-dependently (Supplementary Figure S1). Interestingly, targeting CD38 with daratumumab, an anti-CD38 human antibody showed high single-agent antitumor activity in patients with relapsed and refractory MM (Lokhorst et al., 2015). Therefore, PGG might be an attractive drug for the treatment of CD38-positive MM tumors.

Previously, we reported that PGG inhibits MYC expression both at mRNA as well the protein level in human hepatocellular carcinoma cells (Kant, 2017, unpublished data). The MYC transcription factor is a master regulator of cell proliferation and metabolism (Dang, 2013). Its dysregulation contributes to disease development and progression in a number of human cancers such as blood cancers (Kalkat et al., 2017). In multiple myeloma, MYC is up-regulated and leads to development of the malignant phenotype (Holien and Sundan, 2012; Holien et al., 2012). Therefore, we examined the inhibition of MYC by PGG in MM cell lines. We observed that PGG treatment inhibited MYC expression in all MM cell lines. Furthermore, MYC inhibition induced by PGG was associated with the changes in expression of its target genes (p21, p27, and cyclin D2). Interestingly, our data showed that NCI-H929 cell line is more sensitive to PGG treatment than other two cell lines. Identical sensitivity patterns observed in JQ1 treated MM cells suggested that PGG and JQ1 might act through similar mechanism which is by MYC inhibition in MM.

The mTOR inhibitor protein DEPTOR is often overexpressed in MM patients and its inhibition is reported to result in MM cell death (Peterson et al., 2009). Due to the oncogenic role of DEPTOR in MM, we also investigated the effect of PGG on DEPTOR expression. Our results showed that PGG treatment reduced DEPTOR level in MM cell lines. Collectively, these

results indicated that MYC and DEPTOR inhibition in a part contributed to the growth inhibitory effect of PGG in MM. Moreover, it brings a rationale that PGG could be more effective in patients who express high MYC/DEPTOR activity in clinical settings. Interestingly, in the analysis of microarray results in a GEO database (Supplementary Figure S2), we found that the expression level of DEPTOR is associated with MYC in several types of cancer. An elevated level of DEPTOR can be observed in breast, lung and brain cancer cells with MYC overexpression, while a concomitant decrease in DEPTOR expression was found in MYC depleting cells or in cells treated with BET inhibitors. Therefore, MYC and DEPTOR relationship requires further studies *in vitro* and *in vivo*.

Bortezomib is an FDA-approved proteasome inhibitor drug extensively used for treatment of relapsed myeloma or patients who are not candidates for transplantation (Painuly and Kumar, 2013). Due to the appearance of drug resistance and dose limiting toxicity, various ongoing studies are seeking combination strategy to enhance bortezomib efficacy (Kapoor et al., 2012; Mahindra et al., 2012). MYC inhibition showed synergistic activity with bortezomib in MM cells (Siegel et al., 2015). Therefore, we determined whether MYC inhibitor PGG potentiates the effect of bortezomib in MM cell lines. In accordance with previous findings our results showed that NCI-H929 is most sensitive to bortezomib treatment. Unexpectedly, PGG treatment has an antagonist effect against bortezomib’s growth-inhibiting activity in all tested MM cell lines. Recently, increasing reports found that a vicinal diol group containing natural compounds or antioxidants present in green leaves and green teas can chemically interact with bortezomib and neutralize the effect of bortezomib-mediated cell growth (Jia and Liu, 2013). Because, PGG is a vicinal diol containing natural compound, therefore, the antagonistic effect of PGG on bortezomib might be attributed to its meta-phenolic moieties. These findings further support that vicinal diol dietary supplements should be avoided with bortezomib treatments. In contrast, bortezomib showed no effect on PGG’s anti-myeloma activity. Importantly, PGG did not block the effect of different proteasome inhibitor MG132 mediated cytotoxicity in MM. Therefore, the effect of PGG is specific to bortezomib structure and not universal for all proteasome inhibitors. Moreover, PGG sensitizes the anti-myeloma effect of MG132 in all the MM cell lines tested. To further validate the concept of synergistic anti-myeloma activity of MYC inhibition with bortezomib treatment in our experimental setting, we tested the combination of known MYC inhibitor JQ1 with bortezomib. The combined treatment of JQ1 and bortezomib enhanced cytotoxic effects in the MM cells. Due to drug-drug interaction, PGG cannot be used in combination with bortezomib. On the other hand, it has been shown that PGG is safe and synergizes antitumor effect in various combination treatments (Ryu et al., 2012; Huang et al., 2016; Kant et al., 2016). Therefore, combination effect of PGG with other existing therapies for MM will need to be evaluated in future studies.

In summary, we have shown for the first time the growth inhibitory potential of PGG in multiple myeloma by inducing apoptosis and cell cycle arrest. We demonstrated that PGG inhibited the MYC and DEPTOR expression in myeloma cells. These results support the hypothesis that PGG is a potent MYC inhibitor. However, combination with proteasome inhibitors should be dealt with caution as there may be antagonist effects with specific agents. In conclusion, this study suggested that PGG has a therapeutic potential and warrants further investigation.

## Author Contributions

These studies were conceived of and designed by DT, RK, H-HH, C-HY, and Y-MC. Experiments were performed by DT and RK. Data analysis, data interpretation, manuscript preparation were done by DT, RK, C-HY, H-HH, and Y-MC.

## Conflict of Interest Statement

The authors declare that the research was conducted in the absence of any commercial or financial relationships that could be construed as a potential conflict of interest.
