# Supplementary material for: 1,2,3,4,6-Penta-O-Galloyl-Beta-D-Glucopyranoside Inhibits Proliferation of Multiple Myeloma Cells Accompanied with Suppression of MYC Expression
Source: Front Pharmacol. 2018 Feb 2;9:65. doi: 10.3389/fphar.2018.00065 (PMC5810280; doi:10.3389/fphar.2018.00065)
Supplement: Supplementary file 1 [file Table_1.DOCX]

Supplementary Material

1,2,3,4,6-penta-O-galloyl-beta-D-glucopyranoside Inhibits Proliferation of Multiple Myeloma Cells Accompanied With Suppression of MYC Expression

**Duurenjargal Tseeleesuren ^ǂ^, Rajni Kant ^ǂ^, Chia-Hung Yen *, Hui-Hua Hsiao *, Yi-Ming Arthur Chen***

*** Correspondence:** Professor Yi-Ming Arthur Chen: arthur@kmu.edu.tw; Professor Chia-Hung Yen: chyen@kmu.edu.tw; Dr. Hui-Hua Hsiao: huhuhs@cc.kmu.edu.tw

^ǂ^ These authors contributed equally to this work

# Supplementary Tables

**Supplementary Table S1.** Cell viability assay results for PGG, bortezomib and combination in RPMI 8226 cells.

**Bortezomib
(nM)**

| **PGG (μg/mL)** | **0** | **3.125** | **6.25** | **12.5** | **25** | **50** |
| --- | --- | --- | --- | --- | --- | --- |
| **0** | 100 ± 1.66 | 93.97 ± 4.17 | 89.62 ± 3.56 | 82.03 ± 2.79 | 15.64 ± 1.11 | 8.35 ± 0.47 |
| **3.125** | 93.05 ± 0.84 | 99.53 ± 2.33^*^ | 96.63 ± 1.86 | 91.35 ± 1.22 | 35.4 ± 1.01^***^ | 11.68 ± 0.60^***^ |
| **6.25** | 38.79 ± 1.73 | 100.90 ± 2.00^***^ | 90.99 ± 3.88^***^ | 90.10 ± 3.57^***^ | 37.10 ± 1.16^***^ | 11.20 ± 0.37^***^ |
| **12.5** | -0.09 ± 0.42 | 98.86 ± 0.81^***^ | 99.67 ± 1.16^***^ | 89.60 ± 3.25^***^ | 39.53 ± 1.80^***^ | 11.55 ± 0.20^***^ |
| **25** | -1.22 ± 0.13 | 97.5 ± 1.46^***^ | 97.81 ± 2.77^***^ | 91.78 ± 3.99^***^ | 50.51 ± 1.69^***^ | 11.26 ± 0.85^***^ |
| **50** | -1.17 ± 0.07 | 96.46 ± 4.45^***^ | 96.23 ± 0.87^***^ | 89.12 ± 2.25^***^ | 44.58 ± 1.53^***^ | 12.31 ± 0.51^***^ |

**Supplementary Table S2.** Cell viability assay results for PGG, bortezomib and combination in NCI-H929 cells

**Bortezomib
(nM)**

| **PGG (μg/mL)** | **0** | **3.125** | **6.25** | **12.5** | **25** | **50** |
| --- | --- | --- | --- | --- | --- | --- |
| **0** | 100 ± 3.72 | 111.67 ± 1.81 | 103.47 ± 1.46 | 35.18 ± 1.42 | -0.86 ± 1.07 | -7.06 ± 2.19 |
| **3.125** | 7.27 ± 3.11 | 100.59±4.94^***^ | 97.60 ± 0.99^***^ | 46.02 ± 2.43^***^ | 0.03 ± 0.53^*^ | -6.44 ± 0.79^**^ |
| **6.25** | -10.9 ± 1.80 | 103.47±2.55^***^ | 96.07 ± 3.03^***^ | 41.1 ± 0.65^***^ | 1.37 ± 0.36^***^ | -7.43 ± 0.32^***^ |
| **12.5** | -17.61 ± 1.66 | 99.12 ± 1.95^***^ | 97.12 ± 1.78^***^ | 46.35 ± 3.47^***^ | 1.12 ± 0.94^***^ | -7.32 ± 0.32^***^ |
| **25** | -9.51 ± 0.59 | 93.42 ± 1.28^***^ | 95.79 ± 0.91^***^ | 37.79 ± 3.38^***^ | -0.31 ± 0.52^***^ | -6.86 ± 1.15^***^ |
| **50** | -10.07 ± 0.68 | 6.18 ± 0.74^***^ | 79.00 ± 0.67^***^ | 40.23 ± 1.15^***^ | -0.66 ± 0.73^***^ | -4.76 ± 0.84^***^ |

**Supplementary Table S3.** Cell viability assay results for PGG, bortezomib and combination in U266B1 cells.

**Bortezomib
(nM)**

| **PGG (μg/mL)** | **0** | **3.125** | **6.25** | **12.5** | **25** | **50** |
| --- | --- | --- | --- | --- | --- | --- |
| **0** | 100 ± 6.63 | 102.91 ± 4.02 | 85.27 ± 6.63 | 68.99 ± 3.80 | 52.96 ± 5.38 | 12.85 ± 1.39 |
| **3.125** | 96.31 ± 8.29 | 106.69 ± 3.17 | 84.69 ± 3.03 | 66.47 ± 4.26^***^ | 54.65 ± 1.04^***^ | 14.00 ± 1.05^***^ |
| **6.25** | 74.91 ± 1.39 | 105.33±1.88^***^ | 85.38 ± 5.06^*^ | 66.74 ± 4.91^*^ | 53.89 ± 2.16^***^ | 14.23 ± 1.48^***^ |
| **12.5** | 28.51 ± 2.60 | 105.93±2.63^***^ | 84.16 ± 4.41^***^ | 66.21 ± 3.81^***^ | 53.40 ± 0.79^***^ | 12.52 ± 2.39^**^ |
| **25** | 21.10 ± 2.79 | 105.40±3.05^***^ | 88.88 ± 4.50^***^ | 67.37 ± 2.71^***^ | 53.74 ± 4.24^***^ | 14.69 ± 3.23 |
| **50** | 15.22 ± 0.87 | 100.98±4.97^***^ | 84.35 ± 5.41^***^ | 66.66 ± 2.17^***^ | 53.74 ± 2.77^***^ | 16.21 ± 1.99 |

In each table, MM cells were treated with indicated dosages of mentioned drugs alone and in combinations. After 72 hours cell viability was assessed by the alamarBlue® assay as mentioned in Material and Methods. After background subtraction, cell viability values were normalized to solvent controls and expressed as the percentage of the mean of the relative solvent controls. Each value represents the means ±SD (n=3). Significant differences between bortezomib + PGG and bortezomib alone were indicated by ****P*<0.001, ***P*<0.01; **P*<0.05 (Student's t-test).

**Supplementary Table S4.** Cell viability assay results for PGG, MG132 and combination in RPMI 8226 cells.

**MG132
(μM)**

| **PGG (μg/mL)** | **0** | **3.125** | **6.25** | **12.5** | **25** | **50** |
| --- | --- | --- | --- | --- | --- | --- |
| **0** | 100 ± 5.00 | 88.16 ± 0.91 | 89.46 ± 3.08 | 78.17 ± 1.04 | 27.28 ± 2.64 | 6.15 ± 0.31 |
| **0.0022** | 105.57 ± 1.74 | 89.63 ± 0.79^***^ | 84.66 ± 2.57^***^ | 70.26 ± 2.12^***^ | 21.80 ± 0.69^***^ | 5.55 ± 0.42^***^ |
| **0.009** | 106.67 ± 2.37 | 92.52 ± 3.32^**^ | 85.89 ± 6.56^**^ | 65.61 ± 4.77^***^ | 18.83 ± 0.75^***^ | 5.05 ± 0.15^***^ |
| **0.039** | 107.47 ± 1.31 | 86.62 ± 2.46^***^ | 82.02 ± 3.01^***^ | 66.41 ± 1.25^***^ | 18.70 ± 0.41^***^ | 5.11 ± 0.78^***^ |
| **0.156** | 107.00 ± 2.31 | 89.07 ± 2.16^***^ | 89.37 ± 3.53^**^ | 68.22 ± 1.03^***^ | 11.89 ± 1.68^***^ | 3.79 ± 0.11^***^ |
| **0.625** | -1.32 ± 0.28 | -1.05 ± 0.39 | -1.23 ± 0.16 | -0.63 ± 0.13^*^ | -0.36 ± 0.38^***^ | -0.46 ± 0.28^***^ |

**Supplementary Table S5.** Cell viability assay results for PGG, MG132 and combination in NCI-H929 cells.

**MG132
(μM)**

| **PGG (μg/mL)** | **0** | **3.125** | **6.25** | **12.5** | **25** | **50** |
| --- | --- | --- | --- | --- | --- | --- |
| **0** | 100 ± 2.29 | 101.02 ± 2.89 | 82.61 ± 4.95 | 16.31 ± 2.28 | -4.39 ± 1.71 | -10.46 ± 1.59 |
| **0.0022** | 98.76 ± 4.45 | 102.63 ± 3.73 | 82.09 ± 5.14^***^ | 21.45 ± 3.64^***^ | -4.36 ± 3.49^***^ | -12.27 ±4.03^***^ |
| **0.009** | 96.62 ± 1.96 | 95.08 ± 3.63 | 80.76 ± 2.88^***^ | 21.27 ± 2.43^***^ | -3.30 ± 2.18^***^ | -11.35 ±2.09^***^ |
| **0.039** | 99.53 ± 2.41 | 100 ± 7.20 | 85.95 ± 6.12^**^ | 25.46 ± 4.86^***^ | -2.96 ± 2.39^***^ | -11.25 ±3.35^***^ |
| **0.156** | -1.53 ± 1.55 | -6.45 ± 2.47^**^ | -5.72 ± 3.13^*^ | -11.72 ±3.14^***^ | -12.35 ±2.76^***^ | -18.69 ±2.84^***^ |
| **0.625** | -16.18 ± 2.61 | -12.20 ± 1.10^**^ | -14.06 ± 1.77 | -13.72 ± 1.05^*^ | -15.76 ± 1.60 | -18.86 ± 1.96 |

**Supplementary Table S6.** Cell viability assay results for PGG, MG132 and combination in U266B1 cells.

**MG132
(μM)**

| **PGG (μg/mL)** | **0** | **3.125** | **6.25** | **12.5** | **25** | **50** |
| --- | --- | --- | --- | --- | --- | --- |
| **0** | 100 ± 7.18 | 101.66 ± 4.34 | 78.87 ± 6.00 | 71.95 ± 1.97 | 57.74 ± 2.34 | 26.24 ± 1.89 |
| **0.0022** | 108.96 ± 4.80 | 94.62 ± 2.98^*^ | 85.63 ± 1.24^**^ | 60.19 ± 2.37^***^ | 47.91 ± 1.92^***^ | 24.67 ± 0.87^***^ |
| **0.009** | 105.37 ± 6.67 | 88.91 ± 2.46^*^ | 76.87 ± 4.13^**^ | 60.61 ± 1.46^***^ | 47.83 ± 1.76^***^ | 29.25 ± 1.59^***^ |
| **0.039** | 107.93 ± 4.65 | 95.26 ± 10.88 | 82.33 ± 5.10^**^ | 61.43 ± 1.40^***^ | 51.50 ± 3.27^***^ | 29.09 ± 0.91^***^ |
| **0.156** | 70.93 ± 2.31 | 68.85 ± 5.27 | 58.55 ± 3.61^**^ | 38.04 ± 1.92^***^ | 31.75 ± 1.31^***^ | 19.39 ± 2.10^***^ |
| **0.625** | 11.73 ± 1.05 | 13.62 ± 1.64 | 15.52 ± 1.76^*^ | 14.46 ± 1.28^*^ | 6.75 ± 1.74^*^ | 0.11 ± 0.20^***^ |

In each table, MM cells were treated with indicated dosages of mentioned drugs alone and in combinations. After 72 hours cell viability was assessed by the alamarBlue® assay as mentioned in Material and Methods. Each value represents the means ±SD (n=3). Significant differences between MG132 + PGG and MG132 alone were indicated by ****P*<0.001, ***P*<0.01; **P*<0.05 (Student's t-test).

**Supplementary Table S7.** Cell viability assay results for bortezomib, JQ1 and combination against RPMI 8226 cells.

**Bortezomib
(nM)**

| **JQ1 (nM)** | **0** | **62.5** | **125** | **250** | **500** | **1000** |
| --- | --- | --- | --- | --- | --- | --- |
| **0** | 100 ± 4.52 | 98.26 ± 3.53 | 86.25 ± 3.64 | 70.81 ± 2.90 | 43.30 ± 1.81 | 43.66 ± 1.54 |
| **3.125** | 103.45 ± 3.66 | 99.50 ± 3.50 | 90.77 ± 2.92^***^ | 70.12± 3.31^***^ | 46.22 ± 2.98^***^ | 30.71 ± 2.85^***^ |
| **6.25** | 98.42 ± 2.98 | 97.93 ± 2.73 | 91.26 ± 4.49^**^ | 70.84 ± 2.67^***^ | 47.99 ± 4.01^***^ | 28.87 ± 2.34^***^ |
| **12.5** | 44.04 ± 1.34 | 42.5 ± 2.24 | 37.24 ± 4.59^**^ | 14.55 ± 1.35^***^ | -0.20 ± 0.24^***^ | -0.53 ± 0.34^***^ |
| **25** | -0.81 ± 0.21 | -0.70 ± 0.19 | -0.75 ± 0.22 | -0.79 ± 0.24 | -1.02 ± 0.17 | -1.25 ± 0.25^**^ |
| **50** | -1.39 ± 0.20 | -1.08 ± 0.32 | -1.08 ± 0.23^*^ | -1.14 ± 0.24 | -1.39 ± 0.19 | -1.30 ± 0.17 |

**Supplementary Table S8.** Cell viability assay results for bortezomib, JQ1 and combination against NCI-H929 cells.

**Bortezomib
(nM)**

| **JQ1 (nM)** | **0** | **62.5** | **125** | **250** | **500** | **1000** |
| --- | --- | --- | --- | --- | --- | --- |
| **0** | 100 ± 2.75 | 65.33 ± 1.29 | 45.12 ± 1.11 | 32.14 ± 2.31 | 20.91 ± 2.54 | 15.03 ± 2.04 |
| **3.125** | 75.71 ± 2.63 | 46.31 ± 2.76^***^ | 35.4 ± 2.31^***^ | 22.14 ± 2.01^***^ | 12.93 ± 1.49^***^ | 9.39 ± 2.29^***^ |
| **6.25** | 0.07± 1.65 | -0.21 ± 0.99 | 2.36 ± 1.51^*^ | 3.26 ± 1.60^**^ | 0.56 ± 1.57 | 0.78 ± 1.55 |
| **12.5** | -1.26 ± 1.95 | -0.63 ± 1.55 | -2.36 ± 1.27 | 1.20 ± 1.60^*^ | 0.56 ± 2.16 | 0.21 ± 1.12 |
| **25** | 0.98 ± 1.98 | 1.68 ± 1.72 | 0.28 ± 1.53 | 8.30 ± 1.54^***^ | -1.20 ± 2.29 | -0.57 ± 1.79 |
| **50** | 0.84 ± 2.04 | 1.12 ± 1.77 | 1.00 ± 0.99 | 1.70 ± 1.95 | -4.11 ± 1.99^**^ | -2.69 ± 1.75^**^ |

**Supplementary Table S9.** Cell viability assay results for bortezomib, JQ1 and combination against U266B1 cells.

**Bortezomib
(nM)**

| **JQ1 (nM)** | **0** | **62.5** | **125** | **250** | **500** | **1000** |
| --- | --- | --- | --- | --- | --- | --- |
| **0** | 100 ± 3.23 | 93.49 ± 2.27 | 82.03 ± 2.79 | 66.96 ± 2.60 | 61.34 ± 2.88 | 50.43 ± 1.21 |
| **3.125** | 105.91 ± 2.38 | 89.61 ± 0.88^***^ | 78.89 ± 4.03^***^ | 65.31 ± 2.74^***^ | 54.20 ± 1.52^***^ | 50.67 ± 1.84^***^ |
| **6.25** | 92.45 ± 5.45 | 78.92 ± 2.49^***^ | 72.00 ± 3.00^***^ | 62.90 ± 1.57^***^ | 55.39 ± 2.22^***^ | 50.13 ± 2.32^***^ |
| **12.5** | 54.24 ± 4.95 | 53.02 ± 3.01 | 50.31 ± 1.81 | 46.90 ± 2.09^**^ | 41.89 ± 2.94^**^ | 42.56 ± 1.72^***^ |
| **25** | 21.89 ± 3.29 | 21.77 ± 1.20 | 23.21 ± 1.54 | 26.13 ± 1.26^*^ | 24.90 ± 2.20 | 23.03 ± 1.88 |
| **50** | 18.68 ± 2.54 | 19.71 ± 1.66 | 21.23 ± 1.73 | 24.59 ± 1.71^***^ | 23.63 ± 2.46^**^ | 24.00 ± 1.68^***^ |

In each table, MM cells were treated with indicated dosages of mentioned drugs alone and in combinations. After 72 hours cell viability was assessed by the alamarBlue® assay as mentioned in Material and Methods. Each value represents the means ±SD (n=3). Significant differences between bortezomib + JQ1 and bortezomib alone were indicated by ****P*<0.001, ***P*<0.01; **P*<0.05 (Student's t-test).

**Supplementary Table S10.** Characteristics of multiple myeloma cell lines used in this study.

| Cell lines | Ig Type | CD38 | M-CSF | TNF-α | PCA-1 | Source | PGG (IC_50_) μg/mL | Reference |
| --- | --- | --- | --- | --- | --- | --- | --- | --- |
| U266B1 | IgE | - | - | + | +++ | PB | 34.03 | ([Nilsson et al., 1970](#_ENREF_4); [Gooding et al., 1999](#_ENREF_2); [Zhou et al., 2008](#_ENREF_5)) |
| RPMI 8226 | λ | +/- | +/- | + | +++ | PB | 22.5 | ([Matsuoka et al., 1967](#_ENREF_3); [Gooding et al., 1999](#_ENREF_2); [Zhou et al., 2008](#_ENREF_5)) |
| NCI-H929 | IgA | ++ | ++ | - | ++ | PE | 9.63 | ([Gazdar et al., 1986](#_ENREF_1); [Gooding et al., 1999](#_ENREF_2); [Zhou et al., 2008](#_ENREF_5)) |

Ig, Immunoglobulin; PB, peripheral blood; PE, pleural effusion; TNF-α- tumor necrosis factor-alpha; M-CSF, macrophage-colony stimulating factor; PCA-1, plasma cell-associated antigen-1; PGG, 1,2,3,4,6-penta-O-galloyl-beta-D-glucopyranoside.

**References**

Gazdar, A.F., Oie, H.K., Kirsch, I.R., and Hollis, G.F. (1986). Establishment and characterization of a human plasma cell myeloma culture having a rearranged cellular myc proto-oncogene. *Blood* 67(6)**,** 1542-1549.

Gooding, R.P., Bybee, A., Cooke, F., Little, A., Marsh, S.G., Coelho, E., et al. (1999). Phenotypic and molecular analysis of six human cell lines derived from patients with plasma cell dyscrasia. *Br J Haematol* 106(3)**,** 669-681.

Matsuoka, Y., Moore, G.E., Yagi, Y., and Pressman, D. (1967). Production of free light chains of immunoglobulin by a hematopoietic cell line derived from a patient with multiple myeloma. *Proc Soc Exp Biol Med* 125(4)**,** 1246-1250.

Nilsson, K., Bennich, H., Johansson, S.G., and Ponten, J. (1970). Established immunoglobulin producing myeloma (IgE) and lymphoblastoid (IgG) cell lines from an IgE myeloma patient. *Clin Exp Immunol* 7(4)**,** 477-489.

Zhou, Y., Uddin, S., Zimmerman, T., Kang, J.A., Ulaszek, J., and Wickrema, A. (2008). Growth control of multiple myeloma cells through inhibition of glycogen synthase kinase-3. *Leuk Lymphoma* 49(10)**,** 1945-1953. doi: 10.1080/10428190802304966.
