# Supplementary material for: 1,2,3,4,6-Penta-O-Galloyl-Beta-D-Glucopyranoside Inhibits Proliferation of Multiple Myeloma Cells Accompanied with Suppression of MYC Expression
Source: Front Pharmacol. 2018 Feb 2;9:65. doi: 10.3389/fphar.2018.00065 (PMC5810280; doi:10.3389/fphar.2018.00065)
Supplement: Supplementary file 2 [file Image_1.PDF]

Supplementary Material

1,2,3,4,6-penta-O-galloyl-beta-D-glucopyranoside Inhibits Proliferation of Multiple Myeloma Cells Accompanied With Suppression of MYC Expression

Duurenjargal Tseeleesuren [‡], Rajni Kant [‡], Chia-Hung Yen *, Hui-Hua Hsiao *, Yi-Ming Arthur Chen*

*** Correspondence:** Professor Yi-Ming Arthur Chen: arthur@kmu.edu.tw; Professor Chia-Hung Yen: chyen@kmu.edu.tw; Dr. Hui-Hua Hsiao: huhuhs@cc.kmu.edu.tw

[‡] These authors contributed equally to this work

Supplementary figures

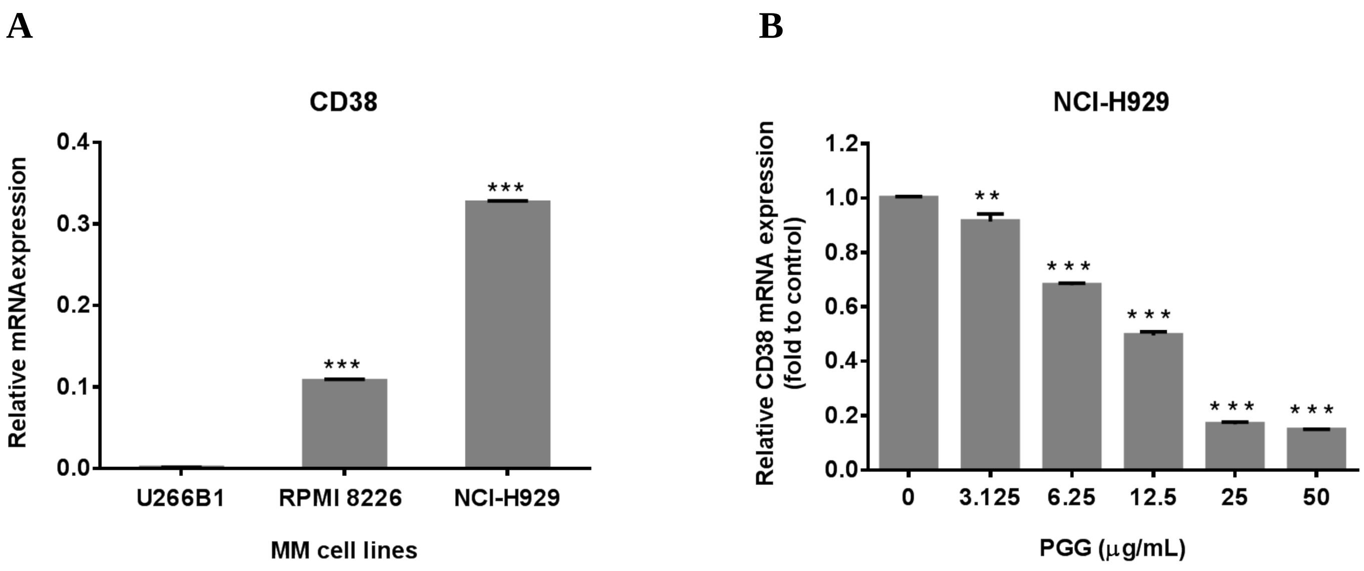

Supplementary Figure S1. PGG inhibits CD38 expression in NCI-H929 cells. **(A)** CD38 mRNA levels in U266B1, RPMI 8226 and NCI-H929 cells were compared directly using qRT-PCR, normalized to TBP mRNA levels. The graph shows the means ± SD (n=3). **(B)** Effect of PGG on CD38 mRNA expression in NCI-H929 cells after 24 hours of treatment. Data presented as fold to solvent control. The graph shows the means ± SD (n=3). ***P<0.001, **P<0.01 (Student's t-test).

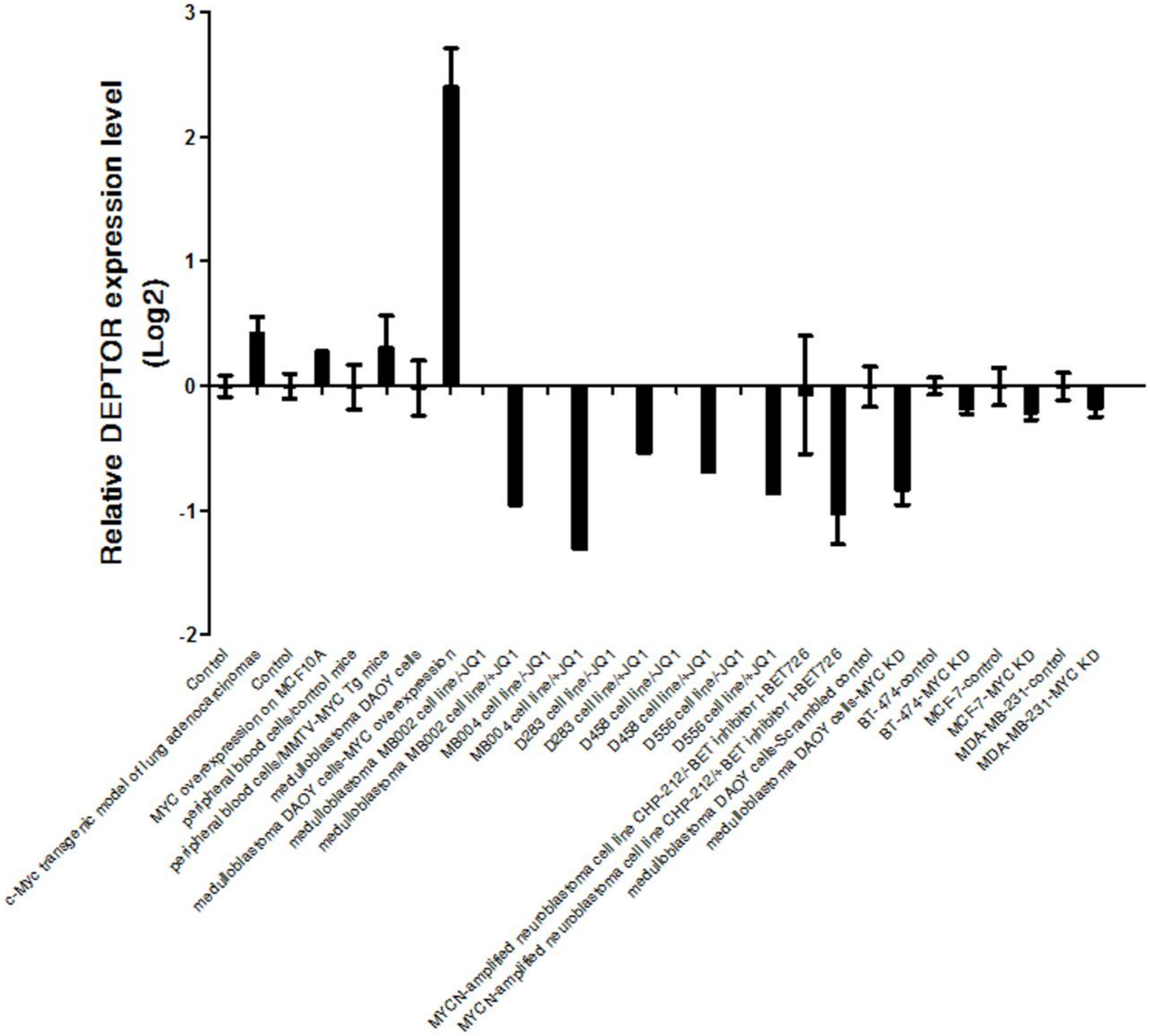

Supplementary Figure S2. A GEO data set shows the positive correlation between c-MYC and DEPTOR expression in different cancer models. In c-MYC overexpressed models such as c-MYC transgenic model of lung adenocarcinoma, c-MYC overexpressed MCF10A, MMTV-MYC transgenic mice and c-MYC overexpressed DAOY cells in medulloblastoma, have higher DEPTOR compared to control. Whereas c-MYC inhibition models such as various medulloblastoma and breast cancer cell lines treated with c-MYC inhibitor or c-MYC knockdown, also shows lower expression of DEPTOR.

Supplementary Figure S3

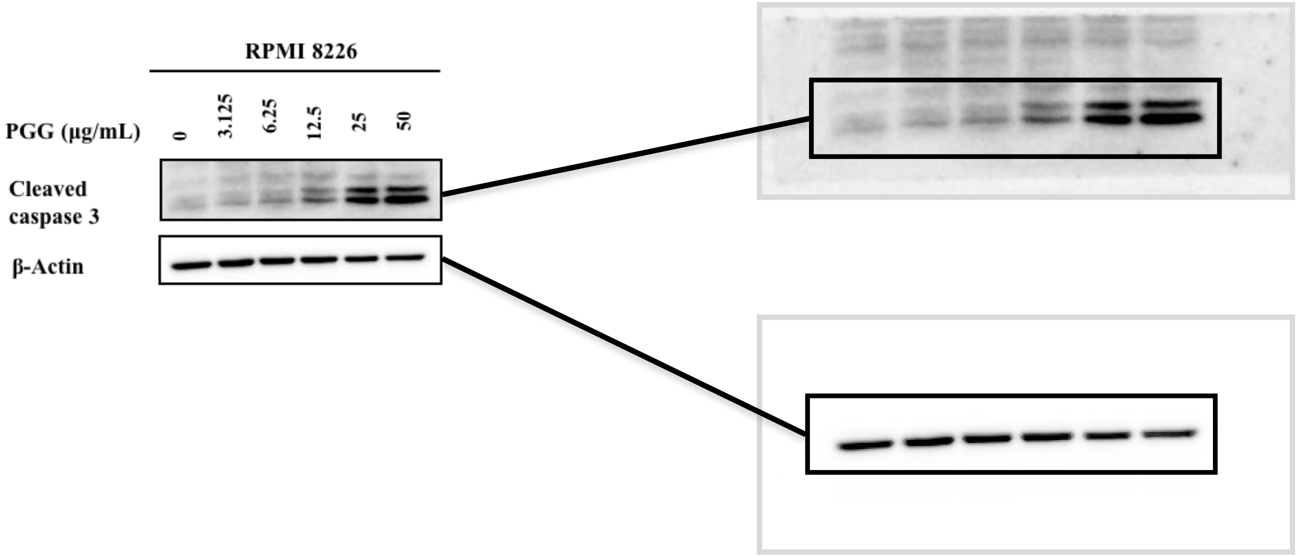

Full unedited image for figure 1G.

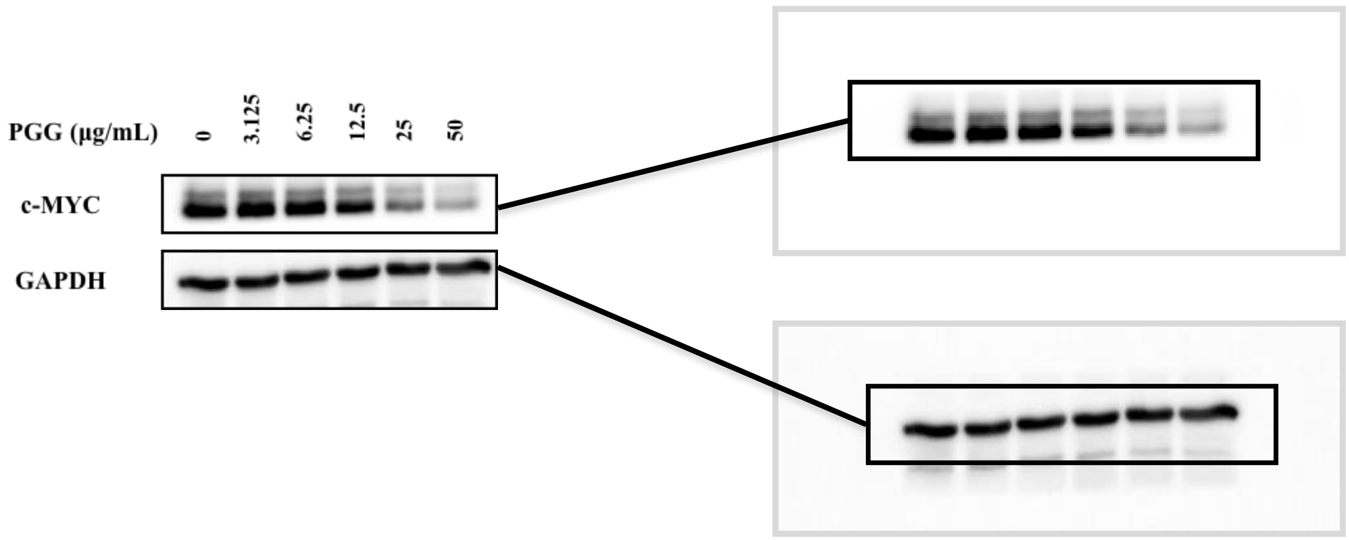

Full unedited image for figure 2B.

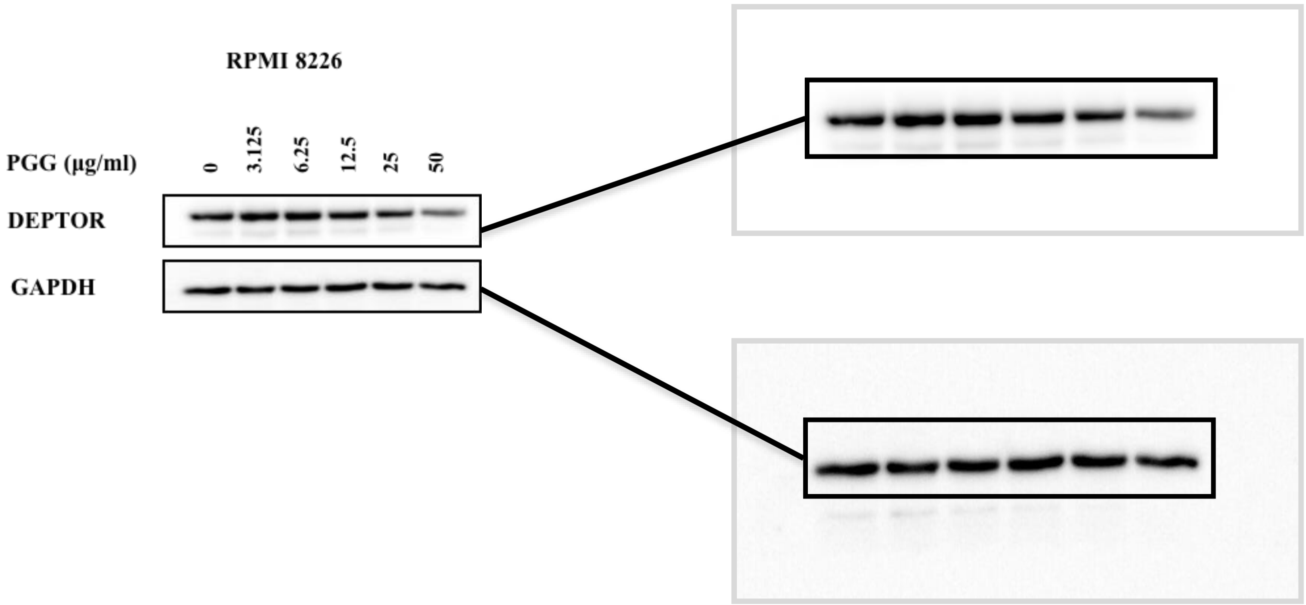

Full unedited image for figure 2E.

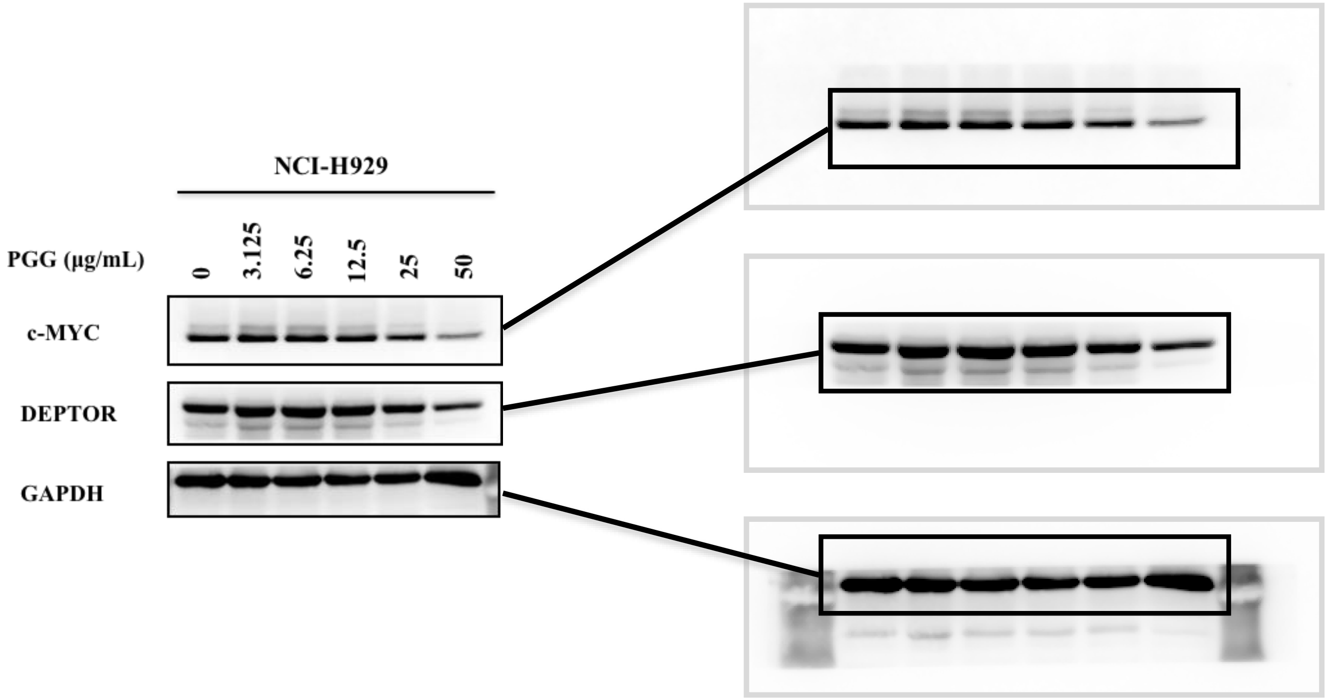

Full unedited image for figure 3B.

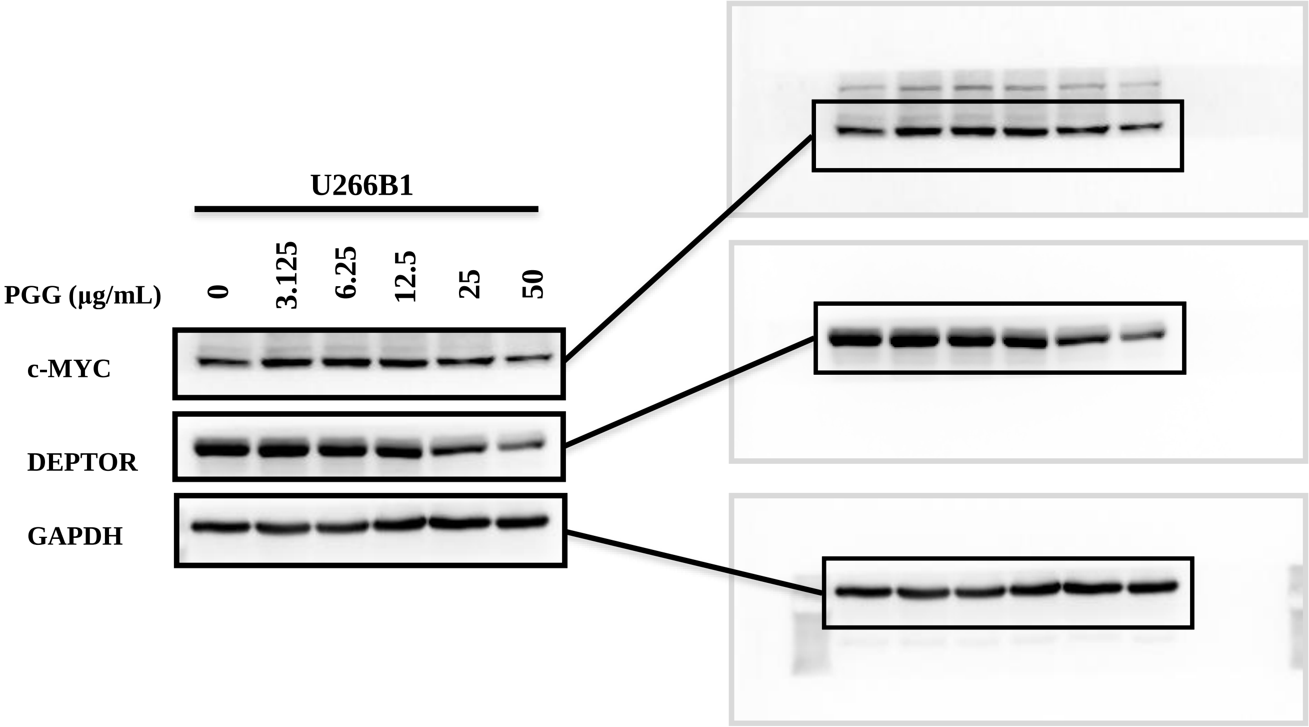

Full unedited image for figure 3C.
